# Cytokines and Lipid Mediators of Inflammation in Lungs of SARS-CoV-2 Infected Mice

**DOI:** 10.3389/fimmu.2022.893792

**Published:** 2022-06-24

**Authors:** Isabelle Dubuc, Julien Prunier, Émile Lacasse, Annie Gravel, Florian Puhm, Isabelle Allaeys, Anne-Sophie Archambault, Leslie Gudimard, Rosaria Villano, Arnaud Droit, Nicolas Flamand, Éric Boilard, Louis Flamand

**Affiliations:** ^1^ Division des maladies infectieuses et immunitaires, Centre de Recherche du Centre Hospitalier Universitaire de Québec- Université Laval, Québec City, QC, Canada; ^2^ Division endocrinologie et néphrologie, Centre de Recherche du Centre Hospitalier Universitaire de Québec- Université Laval, Québec City, QC, Canada; ^3^ Centre de Recherche Arthrite, Université Laval, Québec City, QC, Canada; ^4^ Centre de Recherche de l'Institut universitaire de cardiologie et de pneumologie de Québec, Département de médecine, Faculté de médecine, Université Laval, Québec City, QC, Canada; ^5^ Consiglio Nazionale delle Ricerche, Istituto di Chimica Biomolecolare, Pozzuoli, Italy; ^6^ Département de microbiologie-infectiologie et d'immunologie, Faculté de médecine, Université Laval, Québec City, QC, Canada

**Keywords:** lipid mediators of inflammation, cytokines, chemokines, SARS-CoV-2, COVID-19, pathogenesis

## Abstract

Coronavirus disease 19 (COVID-19) is the clinical manifestation of severe acute respiratory syndrome Coronavirus 2 (SARS-CoV-2) infection. A hallmark of COVID-19 is a lung inflammation characterized by an abundant leukocyte infiltrate, elevated levels of cytokines/chemokines, lipid mediators of inflammation (LMI) and microthrombotic events. Animal models are useful for understanding the pathophysiological events leading to COVID-19. One such animal model is the K18-ACE2 transgenic mice. Despite their importance in inflammation, the study of LMI in lung of SARS-CoV-2 infected K18-ACE2 mice has yet to be studied to our knowledge. Using tandem mass spectrometry, the lung lipidome at different time points of infection was analyzed. Significantly increased LMI included *N*-oleoyl-serine, *N*-linoleoyl-glycine, *N*-oleoyl-alanine, 1/2-linoleoyl-glycerol, 1/2-docosahexaenoyl-glycerol and 12-hydroxy-eicosapenatenoic acid. The levels of prostaglandin (PG) E_1_, PGF_2α_, stearoyl-ethanolamide and linoleoyl-ethanolamide were found to be significantly reduced relative to mock-infected mice. Other LMI were present at similar levels (or undetected) in both uninfected and infected mouse lungs. In parallel to LMI measures, transcriptomic and cytokine/chemokine profiling were performed. Viral replication was robust with maximal lung viral loads detected on days 2-3 post-infection. Lung histology revealed leukocyte infiltration starting on day 3 post-infection, which correlated with the presence of high concentrations of several chemokines/cytokines. At early times post-infection, the plasma of infected mice contained highly elevated concentration of D-dimers suggestive of blood clot formation/dissolution. In support, the presence of blood clots in the lung vasculature was observed during infection. RNA-Seq analysis of lung tissues indicate that SARS-CoV-2 infection results in the progressive modulation of several hundred genes, including several inflammatory mediators and genes related to the interferons. Analysis of the lung lipidome indicated modest, yet significant modulation of a minority of lipids. In summary, our study suggests that SARS-CoV-2 infection in humans and mice share common features, such as elevated levels of chemokines in lungs, leukocyte infiltration and increased levels of circulating D-dimers. However, the K18-ACE2 mouse model highlight major differences in terms of LMI being produced in response to SARS-CoV-2 infection. The potential reasons and impact of these differences on the pathology and therapeutic strategies to be employed to treat severe COVID-19 are discussed.

## Introduction

As of May 2022, the severe acute respiratory syndrome coronavirus 2 (SARS-CoV-2) has infected more than 520 million people worldwide and caused more than 6 million deaths. In addition, the SARS-CoV-2 pandemic led to billions of dollars in economic losses. Fortunately, the rapid development of safe and effective vaccines has limited the spread of the virus, hospitalizations and deaths.

As with all RNA viruses, when given the opportunity to replicate, mutants of SARS-CoV-2 will emerge. This has been observed during the current pandemic with detection of SARS-CoV-2 variants, such as the Delta and Omicron variants that rapidly became the dominant strains as a result of mutations providing increased transmissibility. On occasion, mutations improve viral fitness and/or transmissibility, pathogenicity and reduced neutralization by antibodies generated during previous infection or vaccination are referred to as variants of concern (VOC). According to the Centers for Disease Control and Prevention, the following lineages of SARS-CoV-2 are considered VOC: B.1.1.7, B.1.351, B.1.427, B.1.429, P.1 and B.1.617.2, B.1.1.529.

The study of SARS-CoV-2 pathogenesis, the development of safe and efficacious vaccines and antivirals rely heavily on the use of animal models. Soon after the initial report of SARS-CoV-2 as the causative agent of Coronavirus disease 2019 (COVID-19) (1), several animal models were tested for their susceptibility to infection by this new virus. Unlike humans, hamsters, ferrets and non-human primates do not develop severe disease and these animals generally recover spontaneously. Following the identification of the human Angiotensin Converting Enzyme 2 (hACE2) as a key cellular receptor allowing infectivity of cells by SARS-CoV-2, several animal models initially developed for SARS-CoV that also utilizes ACE2 as receptor were tested. Models ranged from mouse models expressing hACE2 either transiently (through adenoviral vector delivery) (2), from the mouse ACE2 promoter (3, 4) or heterologous promoter such as the lung epithelial cell HFH4 (5) or cytokeratin 18 (K18) promoters (6, 7). Of these models, the HFH4-ACE2 and K18-ACE2 develop severe disease and recapitulate several pathological features observed during severe COVID-19 (8, 9). A recombinant mouse adapted SARS CoV-2 capable of infecting mice through the murine ACE2 receptor was also generated (10). This virus caused more severe damage in older mice.

Work by Winkler et al. (9) and Jiang et al. (8) indicate that ACE2 transgenic mice are highly susceptible to SARS-CoV-2 with detection of virus in several organs with the lungs being the most affected organ. Mice develop pneumonia associated with a decline in pulmonary functions, leukocyte infiltration and presence of several inflammatory cytokines (9). Pre-exposure to SARS-CoV-2 provided protection against a lethal viral challenge (8). Such models should prove useful to the testing of antivirals and vaccines.

Cytokines and chemokines definitely play a crucial role in inflammatory processes. Equally important in the development and resolution of inflammation are Lipid Mediators of Inflammation (LMI), a family of small molecules rapidly generated in response to a variety of stimuli. Some of the most active LMI include leukotrienes, prostaglandins, resolvins, lipoxins and maresins. In recent studies, we analyzed the lung lipidome of human subjects affected by severe COVID-19 and compared it to that of control subjects (11, 12). Bronchoalveolar lavages from severe COVID-19 patients were characterized by increased fatty acids and inflammatory lipid mediators, with a predominance of thromboxane and prostaglandins. Leukotrienes were also increased, notably LTB_4_, LTE_4_, and eoxin E_4_.

In the current study, we aimed at providing a detailed analysis of the LMI produced in response to SARS-CoV-2 infection in K18-ACE2 mice. In parallel to LMI, a detailed analysis of the transcriptome and cytokines/chemokines present in lungs of infected mice was performed. Lastly, similarities and differences in the inflammatory responses observed in SARS-CoV-2 infected human and mice are discussed.

## Materials and Methods

### Cells and Virus

Vero cells were obtained from the American Type Culture Collection (Manassas, Virginia, USA) and cultured in MEM medium supplemented with antibiotics and 10% fetal bovine serum. The SARS-CoV-2 (strain LSPQ, B1 lineage) is similar to the Wuhan strain and contains the following mutations: Spike D614G, NS3 Q57H, NSP2 T85I and NSP12 P323L. The virus was isolated from a 51-year-old female individual from the province of Quebec, Canada by the Laboratoire de Santé Publique du Québec (LSPQ). The virus was propagated on Vero cells. The infectious titer of the viral preparation was 1,8x10^6^ Tissue Culture Infectious Dose 50/mL (TCID_50_/mL).

### Mice

All animal experiments were conducted under biosafety level 3 confinement and approved by the Université Laval animal committee. Nine-week old female B6.Cg-Tg (K18-ACE2)2Prlmn/J (stock# 3034860) (K18-ACE2 mice) were purchased from the Jackson Laboratories (Bar Harbor, ME). Mice were anesthetized using isoflurane and infected by intranasal delivery of 25 ul of saline containing 4.5x10^4^ TCID_50_ of SARS-CoV-2. Mock-treated mice received 25 ul of saline. Mouse body temperature and weight were recorded every day for 7 days. On day 1, 2, 3, 4, 5 and 7, mice (n=5/group) were euthanized, and lungs collected for assessment of viral loads, RNA extraction, tissue homogenization for cytokine lipid mediators of inflammation analysis and histological studies.

### RNA Extraction and RNA-Seq Analysis

RNA from lungs of mice was extracted using the Bead Mill Tissue RNA Purification Kit and the Omni Bead Ruptor Bead Mill homogenizer (Kennesaw, GA). RNA librairies were generated using the NEBNext Ultra II directional RNA library and NEBNext rRNA Depletion kits (New Englang Biolab, Ipswich, MA) and analyzed using the Novoseq 6000 sequencer from Illumina (Vancouver, BC, Canada). This sequencing procedure yielded a total of 703×10^6^ reads, each presenting a length of 100bp. Sequencing quality was assessed for all reads using fastqc0.11.7, and low-quality sequencing as well as remaining adaptor nucleotides were discarded using trimmomatic 0.36 which resulted in a total of 623.2×10^6^ reads of 100bp on average.

The gene expression levels were assessed using the pseudo-alignment approach implemented in Kallisto0.46.1 (13). We applied this method using cleaned reads and a composite reference transcriptome including both murine and SARScov2 coding sequences. Normalizations and statistical tests for differential expressions between time points were performed using the ‘DESeq2’ R-package (14). The principal component analysis and the heatmap were obtained using normalized gene expression levels and the R statistical software. To consider a differential gene expression statistically significant, thresholds of 0.01 and |log_2_ (3)| were applied for the adjusted p-value and the minimum fold-change, respectively (as shown in the Volcano plot).

### Lipid Mediators of Inflammation Analysis

Lung homogenates corresponding to 10 mg of tissues were heated at 60°C for 30 minutes in presence of 1 volume of methanol containing deuterated internal standards. Samples then were processed as in (11) to extract the lipid mediators. The extracts were next analyzed by LC-MS/MS using a previously described analytical method (15).

### Multiplex Cytokine Measurements

The mouse lung cytokine/chemokine contents were assessed using the mouse cytokine/chemokine 31-plex array (Eve technologies, Calgary, Alberta, Canada).

### D-Dimer Measurements

Plasma samples from mock-infected and infected mice were analyzed for the presence of D-Dimers by ELISA according to the manufacturer’s guidelines (Biomatik Corporation, Kitchener, Ontario, Canada).

### Histological and Immunofluorescence Studies

For each mouse analyzed, the right lung was extracted and fixed with formalin. Lungs were paraffin embedded and tissue processed for hematoxylin-eosin staining as described (16).

Immunofluorescence assay for detection of the SARS-CoV-2 N antigen and leukocyte infiltration were performed using rabbit anti-N (Rockland chemicals, Limerick, PA, USA)/anti-rabbit IgG Alexa 488 (Jackson Immuno Research lab, West Grove, PA, USA) and biotinylated anti-CD45 antibodies (BD Bioscience, Franklin Lakes, NJ, USA)/CY3 streptavidin (Thermo Fisher Scientific, Waltham, MA, USA) was as described (17).

## Results

### Infection of K18-ACE2 Mice With SARS-CoV-2

**Figure 1:**
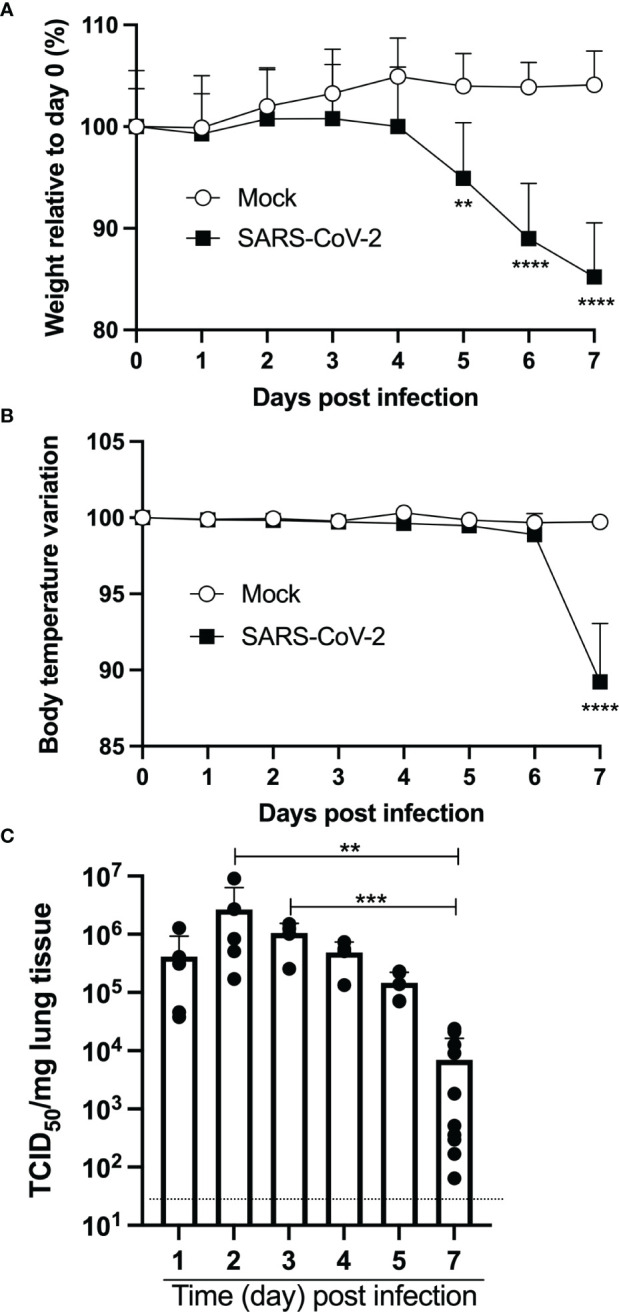
Intranasal infection of K18-ACE2 mice with SARS-CoV-2. Nine-week old female K18-ACE2 mice (n=5-10/group) were inoculated intranasally with 4.5x10^4^ TCID_50_/25ul. Every day for 7 days, weight **(A)**, body temperature **(B)** and lung viral loads **(C)** were monitored. For A and B, two-way ANOVA with Sidak’s multiple comparisons: **p<0.005; ****p<0.0001. For C, ANOVA followed by Kruskal Wallis tests: **p<0.005; ***p<0.001.

Nine-week-old female mice were inoculated intranasally with 4,5x10^4^ TCID_50_ of SARS-CoV-2 (strain LSPQ1). Body temperature and weight were taken daily. The weight of infected mice remained stable over the first 4 days (**Figure 1A**). On day 5 post-infection, SARS-CoV-2-infected mice started to lose weight and by day 7, mice experienced a 15% mean body weight reduction. Body temperature of infected mice remained comparable to that of control mice (36,4 + 0,1˚C) up to day 6. However, a sharp decline in body temperature of infected mice (32,6 + 1,4˚C) was recorded on day 7 post infection (**Figure 1B**), and all infected mice showed lethargy and experienced breathing difficulties and were thus euthanized.

Measurements of lung viral loads (by TCID_50_) indicated very efficient viral replication at early time points sampled (**Figure 1C**). Indeed, maximal viral loads were recorded on days 2 and 3 post-infection. By day 7, viral loads had dropped ≈100-fold and were significantly lower than the maximal viral load observed.

### Lung Histopathology Following SARS-CoV-2 Infection

**Figure 2:**
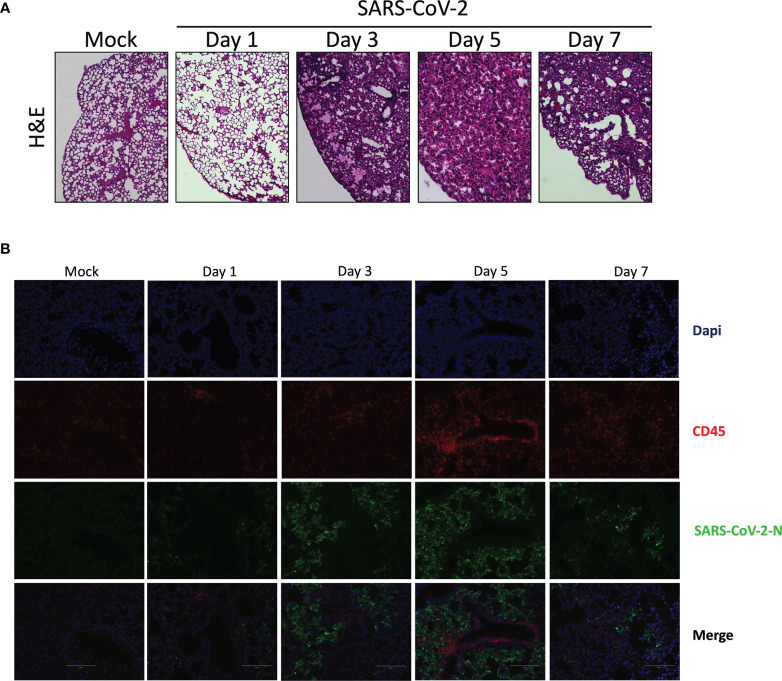
Histological analysis of lungs of mice infected with SARS-CoV-2. Lungs of mock or SARS-CoV-2-infected mice were collected on day 1, 3, 5 and 7 post-infection. Following fixation, lungs were paraffin embedded, sliced and stained with H & E **(A)** or analyzed by immunofluorescence using anti-N (green) and anti-CD45 antibodies (red) to detect SASR CoV-2-infected cells and leukocytes, respectively. **(B)** Nuclei were stained with DAPI. Slides were observed using an Evos M500 microscope.

In contrast to lungs of mock-infected mice that have well defined alveoli and alveolar spaces, lungs of SARS-CoV-2 infected mice displayed pronounced leukocyte infiltration accompanied with thickened alveolar walls and much reduced alveolar spaces (**Figure 2A**). Even though viral replication was robust on day 1 post infection, signs of lung inflammation were scarce. Leukocyte infiltrates were observed starting on day 2 post infection. Such cell infiltrate increased overtime and was maximal at day 7 post infection.

Lung tissues were analyzed by immunofluorescence to detect SARS-CoV-2 infected cells as well as leukocyte infiltration. As shown in **Figure 2B**, a progressive accumulation of SARS-CoV-2 infected cells was detected (**Figure 2B**). Starting on day 3 post-infection we could detect leukocycyte infiltration within several areas of the lungs. Maximal leukocyte infiltrations were recorded at day 5 and 7 post-infection (**Figure 2B**).

### Pro-Coagulation and Thrombotic Events During SARS CoV-2 Infection

**Figure 3:**
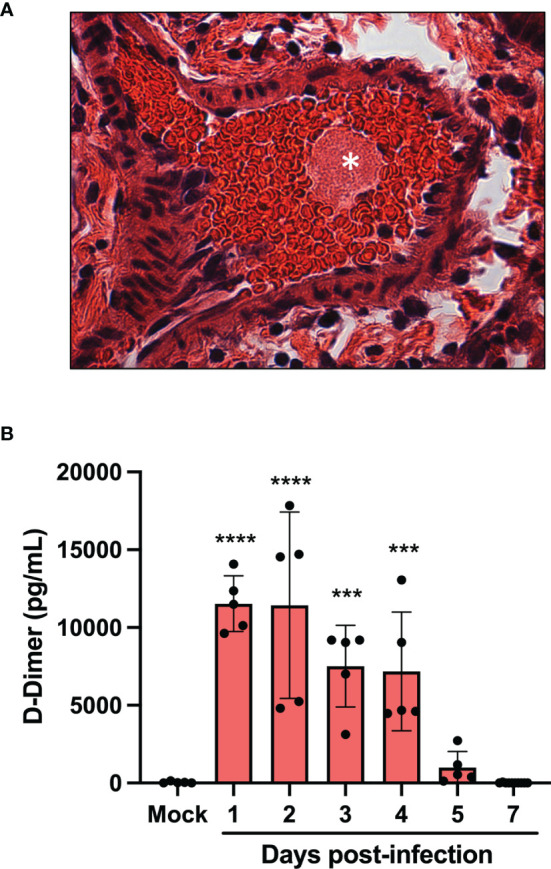
Pulmonary thrombus and D-dimers in plasma of SARS-CoV-2 infected mice. **(A)** Lung tissue section from day 7 SARS-CoV-2-infected showing blood clots (asterisk). **(B)** The concentration of D-dimers in plasma of mock and SARS-CoV-2 infected mice were determined at several time points pos-infection by ELISA. ***p<0.01; ****p<0.0001 as determine by ANOVA followed by Holm-Sidak’s multiple comparisons test.

One of the main medical complications occurring during severe COVID-19 are micro thrombotic events. Histological analysis of the lungs of infected mice revealed the presence of blood clots at late infection times (day 5 and 7) (**Figure 3A**). To prevent blood clots from growing and becoming problematic, a process called fibrinolysis is engaged. Through the action of plasmin, the clot made of fibrin mesh is cut into small fragments. Some of these fibrin degradation products are D-dimers. D-Dimers are generally absent from the circulation unless coagulation processes have been activated. When the plasma levels of SARS-CoV-2 infected mice were analyzed for the presence of D-Dimers, a very significant increase in D-dimer levels were observed at early times (Day 1-4) of infection (**Figure 3B**). These results indicate that soon after infection, SARS-CoV-2 induces the formation of blood clots. In contrast, at late time post infection (day 5 and 7), D-dimer levels were like those of mock-infected mice, suggesting that blot clot formation has ceased or that fibrinolysis of existing blood clots is no longer ongoing.

Analysis of RNA from lungs of mice indicates that several genes encoding factors associated with coagulation are modulated during infection. As presented in **Table 1**, coagulation factor III (tissue factor), factor IX and factor X are significantly upregulated starting on day 3 post-infection. Other genes upregulated include *Plat* (plasminogen activator, tissue) and *Plau* (plasminogen activator, urkinase) whose actions result in the cleavage of plasminogen in plasmin thereby favorizing fibrinolysis. Of interest, expression of genes encoding other plasminogen activators such as Kallikrein and factor XII were downregulated although this difference did not reach statistical significance.

**Table 1:** Relative expression of genes associated with coagulation during SARS-CoV-2 infection.

		SARS-CoV-2 infection							
		**Day 1**		**Day 3**		**Day 5**		**Day 7**	
**Gene ID**	**Gene name**	**Fold log2**	**adj p value**	**Fold log2**	**adj p value**	**Fold log2**	**adj p value**	**Fold log2**	**adj p value**
ENSMUSG00000037411	Serpine1	0,17	8,46E-01	2,75	8,93E-17	1,85	1,12E-07	1,48	1,99E-05
ENSMUSG00000031138	9 F	1,65	N/A	2,39	1,52E-02	1,84	9,39E-02	1,42	1,94E-01
ENSMUSG00000021822	Plau	-0,32	6,90E-01	1,85	9,51E-08	0,99	1,11E-02	0,66	8,79E-02
ENSMUSG00000028128	3 F (TF)	0,24	5,74E-01	1,71	2,20E-17	1,70	8,31E-17	1,85	5,13E-20
ENSMUSG00000031444	10 F	-0,26	6,62E-01	1,25	3,34E-06	0,85	3,63E-03	0,52	8,23E-02
ENSMUSG00000031538	Plat (tPA)	0,26	5,95E-01	0,51	5,20E-02	1,05	1,12E-05	1,96	1,17E-18
ENSMUSG00000031443	7 F	-0,27	5,11E-01	0,28	2,90E-01	0,53	2,56E-02	-0,06	8,34E-01
ENSMUSG00000031196	8 F	0,17	N/A	0,09	9,35E-01	0,45	6,16E-01	-0,56	4,87E-01
ENSMUSG00000109764	Klkb1	0,08	NA	-0,04	9,62E-01	-0,21	7,51E-01	-0,62	2,43E-01
ENSMUSG00000027249	2 F (Thrombin)	-0,21	N/A	-0,61	5,89E-01	0,21	8,58E-01	0,76	3,74E-01
ENSMUSG00000031645	11 F	-0,15	N/A	-0,72	6,32E-01	-1,64	2,51E-01	0,13	9,30E-01
ENSMUSG00000021492	12 F	-0,93	N/A	-1,67	NA	-1,36	NA	0,62	7,47E-01
ENSMUSG00000059481	Plg (Plasminogen)	-0,30	N/A	-2,42	NA	-0,35	NA	-0,51	NA

NA, Not Applicable.

Lastly, out of the coagulation genes analyzed, *Serpin1*, coding for a potent plasminogen activator inhibitor, was the gene most upregulated. These results suggest a pro-coagulation transcriptional activation pattern.

### Transcriptomic Profiling of Genes Expressed in Lungs of SARS-CoV-2 Infected Mice

**Figure 4:**
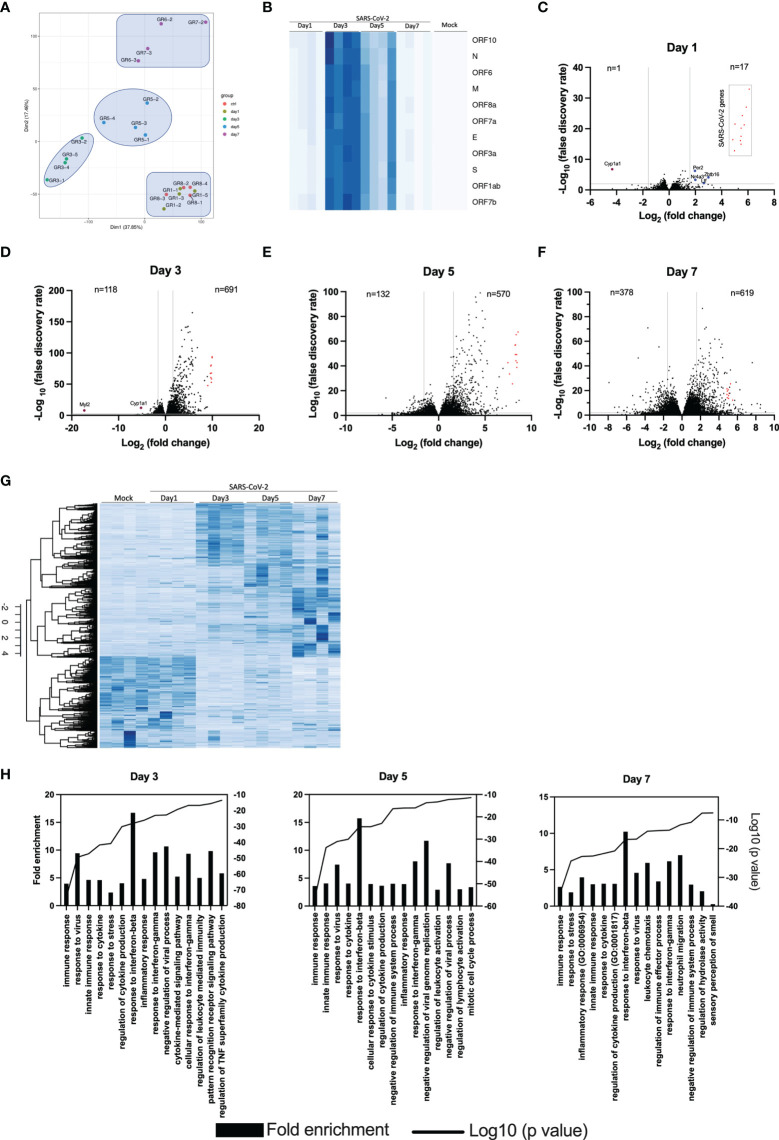
Lung transcriptome analysis of SARS-CoV-2 infected mice. RNA from the lungs of mock and SARS-CoV-2-infected mice (n=4/group) were isolated on days 1, 3, 5 and 7 and subjected to RNA seq. **(A)** Principal component analysis on 20 samples with the normalized gene expression levels. **(B)** Heat maps showing the relative expression of SARS-CoV-2 genes at various times post-infection. The darker the color, the greater the expression. **(C-F)** Volcano plots comparing differentially expressed genes from samples taken at 1, 3, 5 and 7 days post-infection relative to mock-infected mice. Red dots indicate SARS-CoV-2 genes. The numbers in the left and right sections of the volcano plots represent the sum of downregulated and upregulated genes, respectively. **(G)** Heat map representation of cellular genes expression in mock and SARS-CoV-2 infected mice at different times post-infection. The darker the color, the greater the expression. **(H)** Gene Ontology enrichment analysis of biological process enriched from comparisons of mice at day 3, 5 and 7 post infection versus mock-infected mice.

To obtain an overall view of gene modulation during SARS-CoV-2 infection, RNA was extracted from control mice and SARS-CoV-2 infected mice at different time points after infection. Principal component analysis revealed similar signatures between control and infected mice at day 1. In contrast, day 3, 5 and 7 infected mice showed unique transcriptional signatures (**Figure 4A**). Heat map representation of SARS-CoV-2 gene expression indicates that maximal viral transcript expression is detected on day 3 post infection (**Figure 4B**), followed by a gradual decrease at day 5 and 7, which is in accordance with our TCID_50_ data (**Figure 1C**).

Knowing the RNA viruses are subject to mutations, we analyzed the sequence of the viral S gene at day 7 post-infection and compared it to the sequence of the viral inoculum. No mutations were detected (data not shown).

Differential genes expression was gradual, with limited differences on day 1 post infection (**Figure 4C**). In fact, differentially expressed genes were for the most part of viral origin (**Figure 4C**). Starting on day 3 post infection, hundreds of differentially expressed cellular genes were detected with the number of differentially expressed cellular genes continuing to increase up to day 7 post-infection (**Figures 4D–F**). Volcano plots also indicate that the vast majority of differentially expressed genes in infected mice were upregulated rather than downregulated.

Heat map representation of the entire cellular transcriptome indicates minimal variations of gene expression on day 1 post infection. As infection progresses, the number of cellular genes modulated increased with the greatest variations observed at day 7 post infection (**Figure 4G**).

Gene ontology (GO) analysis of top regulated genes in SARS-CoV-2 infected mice indicate enrichment of genes with pathways related to immune response, response to interferons and cytokines and inflammation (**Figure 4H**). On day 7, enrichment of genes associated with granulocyte and neutrophil chemotaxis/migration were also observed. Lastly, genes associated with the sensory perception of smell were downregulated on day 7 post-infection.

**Figure 5:**
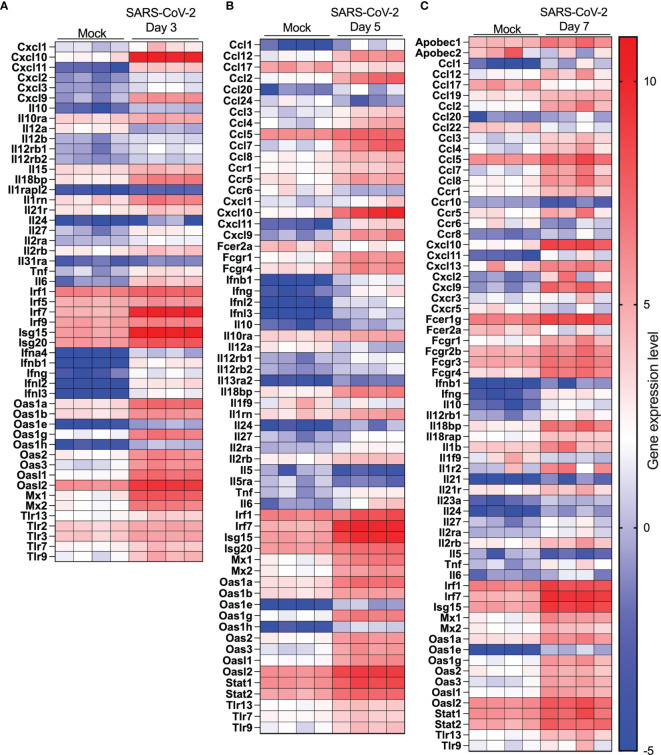
Cytokine and IFN-related gene expression profiles in lungs of SARS-CoV-2 infected mice. RNA from lungs of mock-infected and SARS-CoV-2-infected mice was analyzed by RNA seq. **(A-C)** Heat map representation of genes encoding cytokines/chemokines, IFN or IFN-stimulated genes at day 3 **(A)**, day 5 **(B)** or day (7) post-infection or mock-infected mice.

The top 20 upregulated and downregulated genes at various times post infection are listed under **Supplementary Tables 1**, **2**. Type I, type II or type III interferons (IFNs) as well as CC or CXC chemokines are among the top 20 upregulated genes by SARS-CoV-2 infection (**Supplementary Table 1**). The *Cyp1a1* gene was consistently the most downregulated gene during SARS-CoV-2 infection (**Supplementary Table 2**). Considering that severe COVID-19 is associated with significant pulmonary inflammation, genes associated with host defense and inflammation were analyzed in greater details. As shown in **Figures 5A–C**, several *CC/CXC* chemokines genes as well as antiviral genes, such as *IRF7* and *ISG15*, were among the most upregulated genes throughout infection.

RNA-seq data were also analyzed for modulation in genes encoding enzymes associated with LMI synthesis. As shown in **Table 2**, several genes were significantly upregulated (Pla2g4a, Pla2g4c, Pla2g7, Ptgs1, Ptgs2, Pla1, Tbxas1) or downregulated (Alox5, Pla2g2d, Pla2g1b, Pla2g4b, Pla2g4f) during infection. Our list is however incomplete considering that not all enzymes catalyzing the production of LMI are fully characterized.

**Table 2:** Expression of genes in lungs of SARS CoV-2 infected mice relative to mock infected mice (n=5 mice/group).

	Day 1		Day 3		Day 5		Day 7	
**Gene**	**Log2 Fold**	**adj p Value**	**Log2 Fold2**	**adj p Value3**	**Log2 Fold4**	**adj p Value5**	**Log2 Fold6**	**adj p Value7**
**Alox12**	-0,39	NA	-0,38	2,68E-01	**-0.72187**	**2,13E-02**	-0,31	3,50E-01
**Alox15**	0,95	0,66	-0,34	8,31E-01	-2.03077	8,31E-02	-0,25	8,64E-01
**Alox5**	0,07	0,93	**-1,03**	**6,48E-04**	-0.55169	1,05E-01	-0,65	3,55E-02
**Alox5ap**	-0,36	0,41	0,30	3,12E-01	0.44052	1,19E-01	**1,34**	**6,76E-09**
**Lypla2**	-0,20	0,15	-0,02	8,81E-01	-0.124466	2,45E-01	-0,02	8,74E-01
**Pemt**	0,70	NA	0,13	8,93E-01	0.74134	2,90E-01	0,28	7,08E-01
**Pla1**	0,32	0,72	**2,45**	**8,79E-12**	**2.5652333**	**1,17E-12**	**3,84**	**2,30E-28**
**Pla2g12b**	-0,30	NA	-0,71	6,23E-01	-1.39583	3,45E-01	0,49	6,88E-01
**Pla2g15**	-0,15	0,84	-0,27	5,00E-01	0.2234698	5,89E-01	-0,35	3,22E-01
**Pla2g1b**	-0,43	NA	-0,47	1,08E-01	**-0.77540**	**5,30E-03**	**-1,24**	**1,71E-06**
**Pla2g2c**	-0,16	NA	0,77	5,68E-01	0.201274	9,02E-01	0,24	8,67E-01
**Pla2g2d**	0,47	NA	-0,27	8,33E-01	**-1.9540339**	**3,66E-02**	-0,31	7,82E-01
**Pla2g2e**	-0,88	NA	0,87	3,28E-01	-0.11927	9,23E-01	0,92	2,69E-01
**Pla2g2f**	-22,40	NA	-0,93	NA	-2.49786	NA	-22,28	NA
**Pla2g4a**	0,11	0,85	**0,58**	**1,81E-02**	0.4769864	6,70E-02	**0,71**	**2,31E-03**
**Pla2g4b**	-0,82	NA	-0,20	8,98E-01	-0.712779	5,66E-01	**-2,26**	**1,81E-02**
**Pla2g4c**	0,07	NA	**3,04**	**6,90E-06**	**2.451756**	**6,54E-04**	1,35	7,68E-02
**Pla2g4e**	-1,88	NA	-0,65	7,84E-01	-1.86583	3,42E-01	-0,67	7,48E-01
**Pla2g4f**	-1,32	NA	-0,23	7,79E-01	-0.951797	1,39E-01	**-1,38**	**1,43E-02**
**Pla2g6**	-0,13	0,74	**-0,46**	**1,42E-02**	-0.25338	2,33E-01	-0,11	6,20E-01
**Pla2g7**	0,05	0,93	**1,63**	**1,10E-13**	**1.488195**	**3,12E-11**	**2,94**	**9,27E-44**
**Ptdss1**	-0,16	0,40	-0,07	6,10E-01	-0.15643	2,08E-01	**-0,32**	**1,90E-03**
**Ptgis**	NA	NA	0,21	3,84E-01	0.1415473	5,82E-01	0,33	1,03E-01
**Ptgs1**	0,01	0,98	**0,65**	**4,49E-05**	**0.8115502**	**2,92E-07**	**1,21**	**1,15E-15**
**Ptgs2**	-0,22	0,77	**0,73**	**3,65E-02**	**0.872162**	**1,25E-02**	**-0,67**	**5,20E-02**
**Tbxas1**	-0,22	0,63	-0,04	9,07E-01	0.040398	9,07E-01	**0,57**	**9,23E-03**

NA, Not Applicable. Statistically significant values are in bold.

### Cytokines and Lipid Mediators of Inflammation in Lungs of SARS-CoV-2 Infected Mice

**Figure 6:**
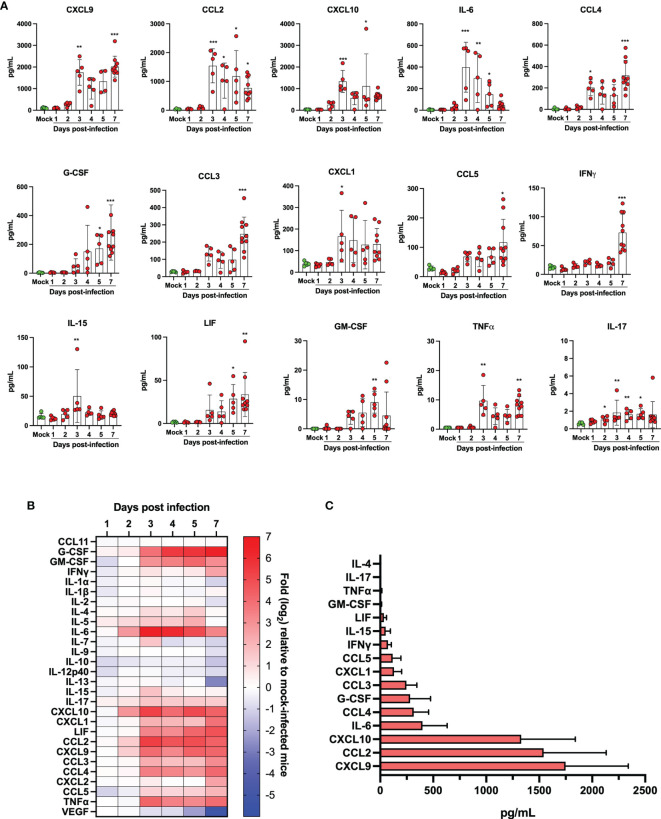
Quantification of cytokine/chemokines in lung homogenates of mock infected and SARS-CoV-2 infected mice. **(A)** Bar graphs representing various cytokine/chemokine concentrations in lung homogenates of mock infected or SARS-CoV-2 infected mice at different times post infection. Data are expressed as mean + SD with each dot representing a mouse. **(B)** Heat map of cytokine/chemokine levels as measured using multiples platform in lungs of mock and SARS-CoV-2 infected mice at various time points post-infection. Results are expressed as fold expression relative to mock infected mice. **(C)** Maximal absolute concentration of selected cytokines/chemokines in lungs of SARS-CoV-2 infected mice. Bars represent the mean +SD of the highest cytokine/chemokine concentration detected at any given time during infection. *p<0.01, **p<0.01, ***p<0.001.

The lungs of SARS-CoV-2 infected subjects that develop severe COVID-19 are filled with several cytokines and lipid mediators of inflammation (LMI) (11, 12, 18, 19). To determine whether a similar inflammatory signature was observed in lungs of SARS-CoV-2 infected mice, the cytokine and LMI contents of lung homogenates were analyzed. The concentration of certain cytokines, including CCL11, IL-1β, IL-5 and CXCL2 were similar between infected and control mice throughout the course of infection (not shown). Others, such as G-CSF, GM-CSF, IFNγ, IL-6, IL-15, IL-17, CXCL1, CXCL9, CXCL10, LIF, CCL2, CCL3, CCL4, CCL5 and TNFα were significantly increased in SARS-CoV-2 infected mice relative to control mice (**Figures 6A, B**). Others, such as IL-1α, IL-2, IL-7, IL-9, IL-10, IL-12p40, IL-13, and VEGF were present at equivalent concentrations in lungs of infected and control mice during the first 5 days, but at significantly reduced concentrations in lungs of infected mice at day 7 post-infection (**Figure 6B**). When the maximal absolute concentration at any given point was examined, the three most abundant cytokines produced in response to SARS-CoV-2 infection in the lungs were CXCL9, CCL2 and CXCL10, respectively (**Figure 6C**).

**Figure 7:**
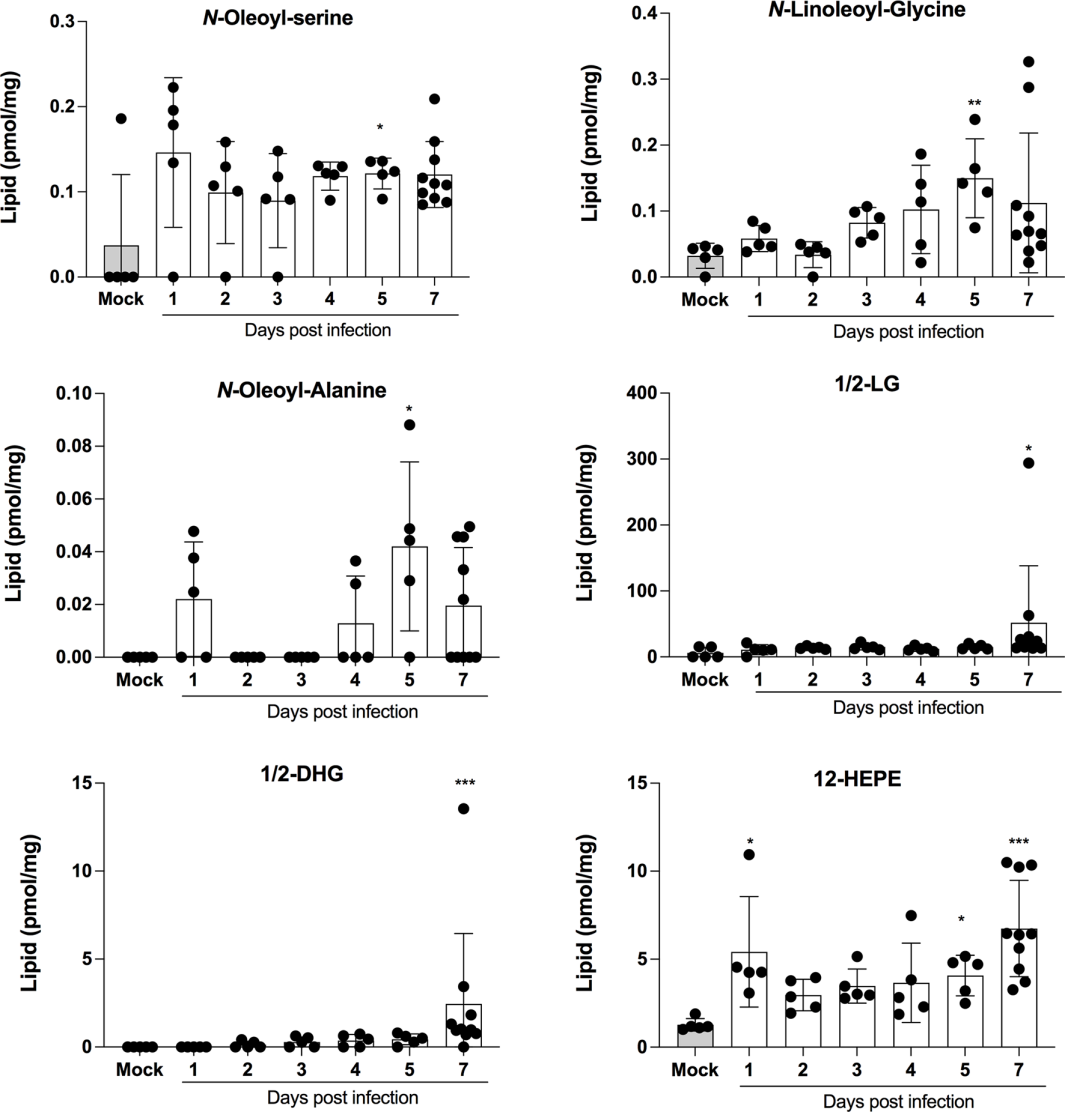
Lipid mediators that are increased in lungs of SARS-CoV-2 infected mice. Lung homogenates corresponding to 10 mg of tissues were heated at 60˚C for 30 minutes in presence of 1 volume of methanol containing deuterated internal standards. Samples then were processed as in Archambault et al (11) to extract the lipid mediators and were analyzed by LC-MS/MS using a previously described analytical method (15). P values were determined using the Kruskall-Wallis test, with p values <0.05 considered statistically significant. *p<0.05, **p<0.01, ***p<0.001.

**Figure 8:**
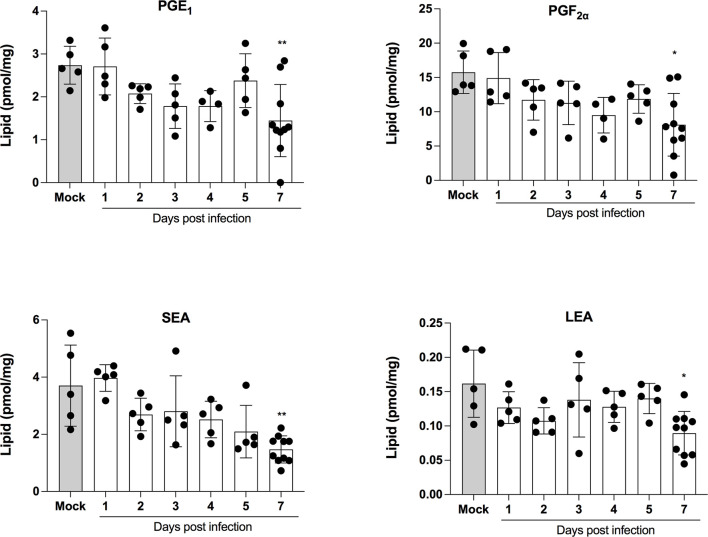
Lipid mediators that are decreased in lungs of SARS-CoV-2 infected mice. Lung homogenates corresponding to 10 mg of tissues were heated at 60˚C for 30 minutes in presence of 1 volume of methanol containing deuterated internal standards. Samples then were processed as in Archambault et al (11) to extract the lipid mediators and were analyzed by LC-MS/MS using a previously described analytical method (15). P values were determined using the Kruskall-Wallis test, with p values <0.05 considered statistically significant. *p<0.05, **p<0.01.

LMI represent another important class of inflammatory mediators. Recent studies indicate that eicosanoids and docosanoids are present at significantly higher concentrations in bronchoalveolar fluids of severe COVID-19 patients than control subjects (11, 12). Lung homogenates were therefore analyzed for LMI content. Significantly increase LMI included *N*-oleoyl-serine, *N*-linoleoyl-glycine, *N*-oleoyl-alanine, 1/2-linoleoyl-glycerol (LG), 1/2-docosahexaenoyl-glycerol (DHG) and 12-hydroxy-eicosapenatenoic acid (12-HEPE) (**Figure 7**). The levels of prostaglandin (PG) E_1_, PGF_2α_, stearoyl-ethanolamide (SEA) and linoleoyl-ethanolamide (LEA) were found to be significantly reduced relative to mock-infected mice (**Figure 8**). The levels of all other detected LMI were not different than those observed in lungs of control non-infected mice (**Supplementary Figure 1**). The fact that *Alox5* gene was downregulated during infection may account for the lack of detection of leukotrienes. Considering the increased expression in phospholipase A2 genes (*Pla2g4a, Pla2g4c and Pla2g7*) and prostaglandin synthase genes (*Ptgs1 and Ptgs2*), increased synthesis of mediators such as prostaglandins was expected. On the contrary, PGE1 and PGF2a levels were below those on control mice (**Figure 8**) while those of PGE2 were similar to controls (**Supplementary Figure 1**). As a result, no correlations between gene expression and LMI synthesis were noted.

**Figure 9:**
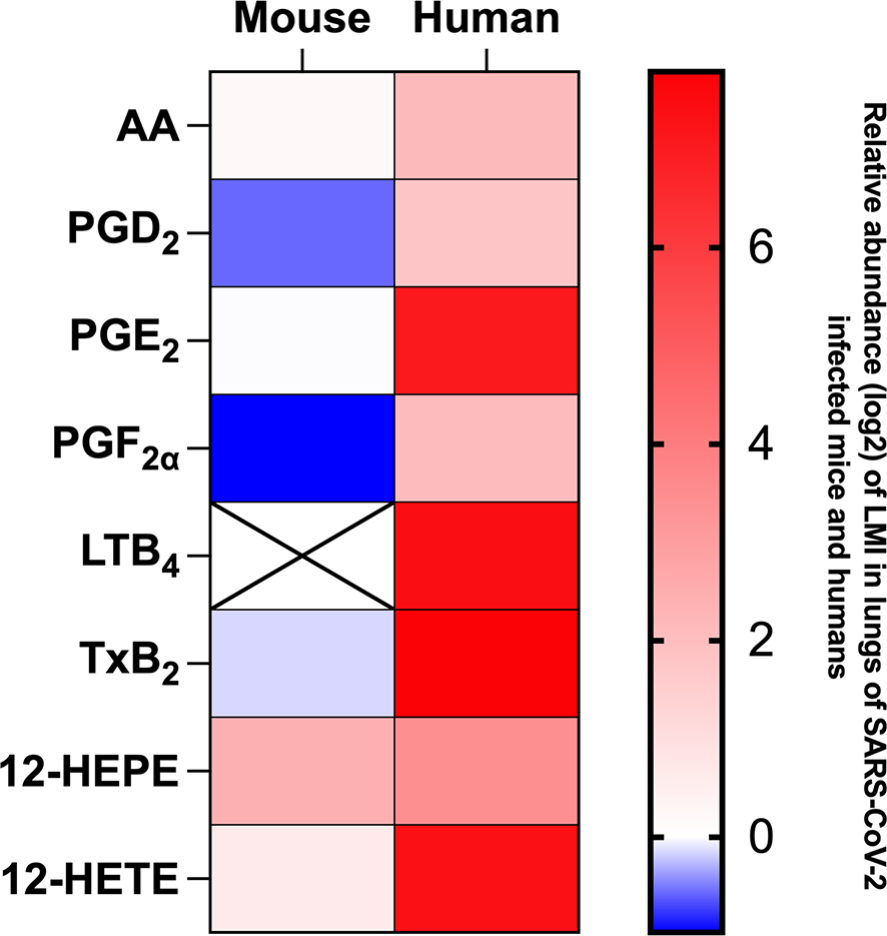
Heat map representation of LMI detected in mouse lung homogenates on day 7 of infection and in bronchoalveolar lavages of hospitalized human patients during severe COVID-19. Results are expressed as log_2_ concentration (pg/mL) relative to LMI detected in lung homogenates of mock-infected mice of healthy human subjects.

Measure of LMI in lung homogenates of SARS-CoV-2 infected K18 ACE2 mice differ considerably that results we obtained in broncho alveolar lavages (BAL) of subjects affected by severe COVID-19 (11, 12). For comparison purpose, a heat map representing the detection of certain lipids relative to mock-infected mice or human control subjects (**Figure 9**). As observed, the LMI content in lungs of infected mice differs minimally from those of control mice. In sharp contrast, BAL of subjects affected with severe COVID-19 contain significantly higher levels of LMI relative to controls (11, 12). These results highlight a major and possibly important difference in the pathophysiology of COVID-19 between humans and mice.

## Discussion

Animal models that recapitulate human diseases are essential to study pathogenesis and useful for the development of effective antiviral strategies and vaccines. The current study provides valuable information by detailing the course of infection of K18-ACE2 mice by SARS-CoV-2. During the first 4 days, clinical signs of infection were non-apparent with no weight loss or temperature fluctuations observed. It is during this period however that viral loads and inflammatory cytokine productions in the lungs were maximal. Starting on day 5, gradual and significant weight losses were recorded in infected mice. By day 7, mice had lost 15% of their initial body weight and experienced a sudden drop in body temperature accompanied with breathing difficulties and lethargy. At this point, mice were euthanized. These results indicate that SARS-CoV-2 readily infects K18-ACE2 mice through the nasal cavity without prior adaptation and causes severe disease. Our results are therefore in agreement with the work of several laboratories (20–23). Our work also argues that in response to early vigorous viral replication in the lungs, a robust inflammatory response is initiated. Such a response, characterized by the release of several chemotactic and inflammatory cytokines/chemokines results in leukocyte migration into the lung parenchyma resulting in oedema and congestion of the alveoli that culminates in acute respiratory disease syndrome (ARDS). We and other have recently reported that human lungs of severe COVID-19 patients contain high levels of chemokines (12, 19, 24–26). In fact, of the 13 most abundant mediators in BAL fluid of severe COVID-19 subjects, 11 were chemokines, with CXCL1 and CXCL8 being 200 times more abundant than IL-6 and TNF-α (12). Similarly, the three most abundant inflammatory mediators in lungs of SARS-CoV-2 infected mice were CXCL9, CCL2 and CXCL10, all three belonging to the chemokine superfamily. These results suggest that SARS-CoV-2 infection of humans and mice triggers lung inflammation through the induction of chemokines. Of interest and potential importance, we reported that subjects with severe COVID-19 also had elevated levels of several LMI, including eicosanoids, with a predominance for thromboxane and prostaglandins arguing that these mediators are also likely contributors of inflammation (11). Using the same sample processing and analytical method as well as the same analytical instrument, very little modulation of LMI was observed in the lungs of mice after SARS-CoV-2 infection (**Figures 7**, **8** and **Supplementary Figure 1**). Importantly, the extraction of our internal standards was similar for both human and mouse matrices arguing that the observed differences are not the consequence of a differential extraction of LMI. Among the LMI that were elevated, the most interesting trends were noticed with monoacylgycerols and *N*-Acyl-ethanolamines. While monoacylglycerols had a tendency to increase at day 7 (reaching significance for 1/2-LG and 1/2-DHG) *N*-Acyl-ethanolamines tended to decrease (reaching significance for LEA and SEA). Monoacylglycerols and *N*-Acyl-ethanolamines are part of the endocannabinoidome and can thus affect multiple receptors (27). Consequently, the opposite effect of infection on the two classes of LMI is difficult to explain at this time as the final outcome involves too many cell receptors. Moreover, some prostaglandins were also decreased at day 7. Some might argue that this could be related to decreased eicosanoid biosynthetic enzymes. To that end, expression profiling indicates that many *Pla2* genes involved in the generation of eicosanoids responsible for the generation of arachidonic acid, the precursor of leukotrienes and prostaglandins, *Alox5* encoding 5-lipoxygenase that participates in the generation of leukotrienes and *Ptgs1* and *Ptgs2*, essential for the generation of prostaglandins, were modestly modulated or significantly downregulated in lungs of SARS-CoV-2 infected mice. We cannot exclude that other biosynthestic enzymes are also modulated (e.g. PGF_2α_ synthase). Furthermore, we cannot eliminate the possibility that the increase of LMI that were observed in humans occurs subsequent to the cytokine/chemokine storm and that perhaps the course of the disease in mice occurs too rapidly to allow the robust increase in LMI observed in humans. Unfortunately, the health status of infected mice prevented us to investigated longer time points to verify this hypothesis. It should be noted that for human samples, BAL fluids were analyzed while for mice, lung homogenates were utilized. Another possible explanation is the age of the animals. Our experiments were conducted with 9-week old mice. It will be relevant to repeat these studies using much older mice to determine whether old age is related to increased LMI production in response to SARS-CoV-2. An important consideration is the fact that laboratory mice have much less neutrophils relative to humans. Knowing that neutrophils are a recognized and important source of LMI, notably eicosanoids, such a difference may explain, at least in part, the observed results. Importantly, the lack of a robust increase in LMI in mice underscores that the investigation of LMI, their biosynthetic enzymes and their receptors in SARS-CoV-2-infected mice might not reflect their involvement in human infection and should thus be interpreted with caution.

Differential gene expression analysis reveals minor differences between control and one day old SARS-CoV-2-infected mice. In fact, the PCA analysis cannot discriminate between these two groups despite SARS-CoV2 genes being highly expressed. This suggest that at early times of infection, the immune response against SARS-CoV-2 is very limited, allowing the virus to replicate and remain largely undetected. Only 5 cellular genes (*Cyp1a1, Ltf, Nr4a3, Per2 and Zbtb16*) were significantly modulated on day 1 post-infection. Of potential interest, the *Cyp1a1* gene was consistently among the most downregulated gene throughout the course of SARS-CoV-2 infection. Cyp1a1 is a member of the cytochrome P450 superfamily of proteins whose functions are not entirely defined and whether such modulation of Cyp1a1 is associated with disease remains to be examined. The overall gene expression profiles changed considerably at 3, 5 and 7 days post-infection with each time point clearly distinguishable from each other using PCA analysis. Our results differ somewhat with those of Winkler et al. (9) that observed overlapping transcriptional patterns up to 4 days post-infection. Our results show that mice infected for 24h have transcriptional patterns identical to mock-infected mice while mice infected for 3, 5 or 7 days have distinct transcriptional patterns. A slightly higher viral inoculum used in our study may account for such differences. The number of positively modulated genes varied between 570 and 691 while the number of downmodulated genes increased with time of infection varying from 118 at day 3 to 378 at day 7 post infection. Several cytokines/chemokines and antiviral genes such as IFNs or IFN-stimulated genes were in the top 20 modulated genes during infection. *Irf7*, a key gene involved in the regulation of IFNα was strongly upregulated (5.3 log2) at day 5 post-infection (p=2,71x10^-281^) but none of the IFNα genes were. This observation can be explained by the fact that SARS-CoV-2 Nsp1 protein inhibits translation of cellular mRNA. Thus, despite strong *Irf7* gene activation, IRF7 is made much less abundantly and therefore cannot efficiently activate IFNα genes. IFNα are also produced by specialized cells such as plasmacytoid dendric cells and leukocytes that may have not been present in sufficient numbers in lungs of infected mice for detection. Other IFNs, such as IFNβ and IFNΛ, are regulated differently, mostly through IRF3/NF-κB, which are constitutively expressed and regulated by post-translational event such as phosphorylation. IFNβ and IFNα are also produced by several cell types including target cells of infection.

Patients affected by severe COVID-19 have a high incidence of thrombosis with a high burden of pulmonary and systemic platelet-rich microthrombi (28–32). Others and we have reported the platelets from SARS-CoV-2 infected patients display a highly activated phenotype, show hyperresponsiveness to thrombin and are responsible, at least in part, for the increase concentration of inflammatory and coagulation mediators (33–36). Thus, activated platelets may contribute to both the overwhelming inflammation and thrombosis that prevail in COVID-19. Thrombi in lungs of mice infected with SARS-CoV-2 were also detected indicating coagulation issues. RNA-seq analysis does suggest that pro coagulation conditions are favored. This includes overexpression of Serpin1 encoding for a plasminogen activator inhibitor, when elevated represents a rick for thrombosis (37) as well as downregulation of kallikrein and factor XII, two plasminogen activators. Tissue factor is another pro coagulating agent likely to play a role in thrombosis during COVID-19. TF is expressed by damaged endothelial cells or exposure to inflammatory cytokines (38). Considering that endothelial cells can be infected by SARS-CoV-2 (39) and that multiple inflammatory cytokines are produced in response to infection, the observation that TF expression is induced during SARS-CoV-2 infection is not without surprise. In fact, a recent report further indicates that TF is released during SARS-CoV-2 infection, triggering platelet activation in the presence of residual amounts of plasma (40). D-dimer concentration in blood was maximal on day 1 post-infection suggesting that clotting issues were occurring very soon after infection. Elevated D-dimer levels in humans infected with SARS-CoV-2 were reported in a number of studies (36, 41–44). Our results suggest that at early times after infection, fibrinolysis of blot clots was occurring efficiently as demonstrated by the presence of D-dimers in plasma. However, at late time points D-dimer plasma concentration fell dramatically suggesting that clots were no longer efficiently resolved, as evidenced by the detection of blood clots in lungs of infected mice. Low fibrinolysis is therefore likely associated with ARDS. Thrombolytic therapy may therefore prove beneficial in severe COVID-19. In support, a clinical phase 2 trial (NCT04357730) testing the efficacy of Alteplase bolus followed by full anticoagulation demonstrated significant and sustained improvements in Pao_2_/Fio_2_ and suggests that clinically relevant endpoints such as ventilator free days and mortality rates could also be improved for patients with severe COVID-19 (45).

Despite its usefulness, this model has its limitations. Expression of the ACE2 receptor is driven from a keratin promoter, meaning that tissue distribution is not the same as that of naturally expressed ACE2. Furthermore, the human *ACE2* gene is located on the X chromosome while several copies of the transgene are inserted in the mouse chromosome 2. Another limitation to our study is the fact that only female mice were studied and that certain LMI such as leukotrienes and prostaglandins are produced differently according to the sex of the individual (46–48). Lastly, comorbidities are known to play significant roles in the severity of SARS-CoV-2 infection. Such comorbidities cannot readily be reproduced in such a mouse model. Despite these limitations, the K18-ACE2 model are useful for the testing of antiviral and vaccine candidates as well as for understanding the pathophysiology of SARS-CoV-2 infection (9, 49, 50).

In summary, we provide evidence that the K18-ACE2 mouse model recapitulates several of the pathologic features observed in human subjects suffering with severe COVID-19, including severe lung inflammation, thrombi formation and ARDS. Our results indicate that intranasal delivery of SARS CoV-2 to K18-ACE2 mice results in severe disease characterized by high viral loads at early time points that were associated with elevated plasmatic D-dimer concentrations suggestive of coagulation events occurring at early times (days 1 and 2) after infection. Pulmonary leukocyte infiltration and secretion of inflammatory mediators starting at day 3 post infection resulted in lung congestion and respiratory failure. Transcriptomic analysis of infected lung tissue reveals that hundreds of cellular genes are modulated progressively throughout infection. This model are useful for the study of SARS-CoV-2 pathogenesis, antiviral and vaccine development.

## Data Availability Statement

The data presented in the study are deposited in the Gene Expression Omnibus repository, accession number GSE205014.

## Ethics Statement

The animal study was reviewed and approved by Comité de protection des animaux de l’Université Laval.

## Author Contributions

ID, ÉL, AG, FP, IA, AS-A, RV, LG performed experiments. JP, AD, NF, ÉB, LF analyzed the data. NF, ÉB and LF conceived the study. LF wrote the paper. All authors reviewed the paper. All authors contributed to the article and approved the submitted version.

## Funding

This work was supported by grants from the Canadian Institutes of Health Research CIHR (#VR3_172632) awarded to LF (PI), ÉB (co-PI) and NF (co- PI) and Fondation du CHU de Québec (Fond Bio-Expert) awarded to LF and by the Coronavirus Variant Rapid Response Network (CoVaRR-Net). ÉL is the recipient of the Fondation du CHU de Québec-UL scholarship, FP is recipient of a Fonds de Recherche du Queíbec en Santé (FRQ-S) fellowship and AS-A received a doctoral award from CIHR. EB is recipient of a senior scholar from FRQ-S.

## Conflict of Interest

The authors declare that the research was conducted in the absence of any commercial or financial relationships that could be construed as a potential conflict of interest.

## Publisher’s Note

All claims expressed in this article are solely those of the authors and do not necessarily represent those of their affiliated organizations, or those of the publisher, the editors and the reviewers. Any product that may be evaluated in this article, or claim that may be made by its manufacturer, is not guaranteed or endorsed by the publisher.

## Supplementary Material

Supplementary Figure 1 Main lipid mediators that are unchanged in lungs of SARS-CoV-2 infected mice. Lung homogenates corresponding to 10 mg of tissues were heated at 60˚C for 30 minutes in presence of 1 volume of methanol containing deuterated internal standards. Samples then were processed as in Archambault et al (11) to extract the lipid mediators and were analyzed by LC-MS/MS using a previously described analytical method (15). P values were determined using the Kruskall-Wallis test, with p values <0.05 considered statistically significant.
