# Supplementary figures and images for: Cytokines and Lipid Mediators of Inflammation in Lungs of SARS-CoV-2 Infected Mice
Source: Front Immunol. 2022 Jun 24;13:893792. doi: 10.3389/fimmu.2022.893792 (PMC9264370; doi:10.3389/fimmu.2022.893792)

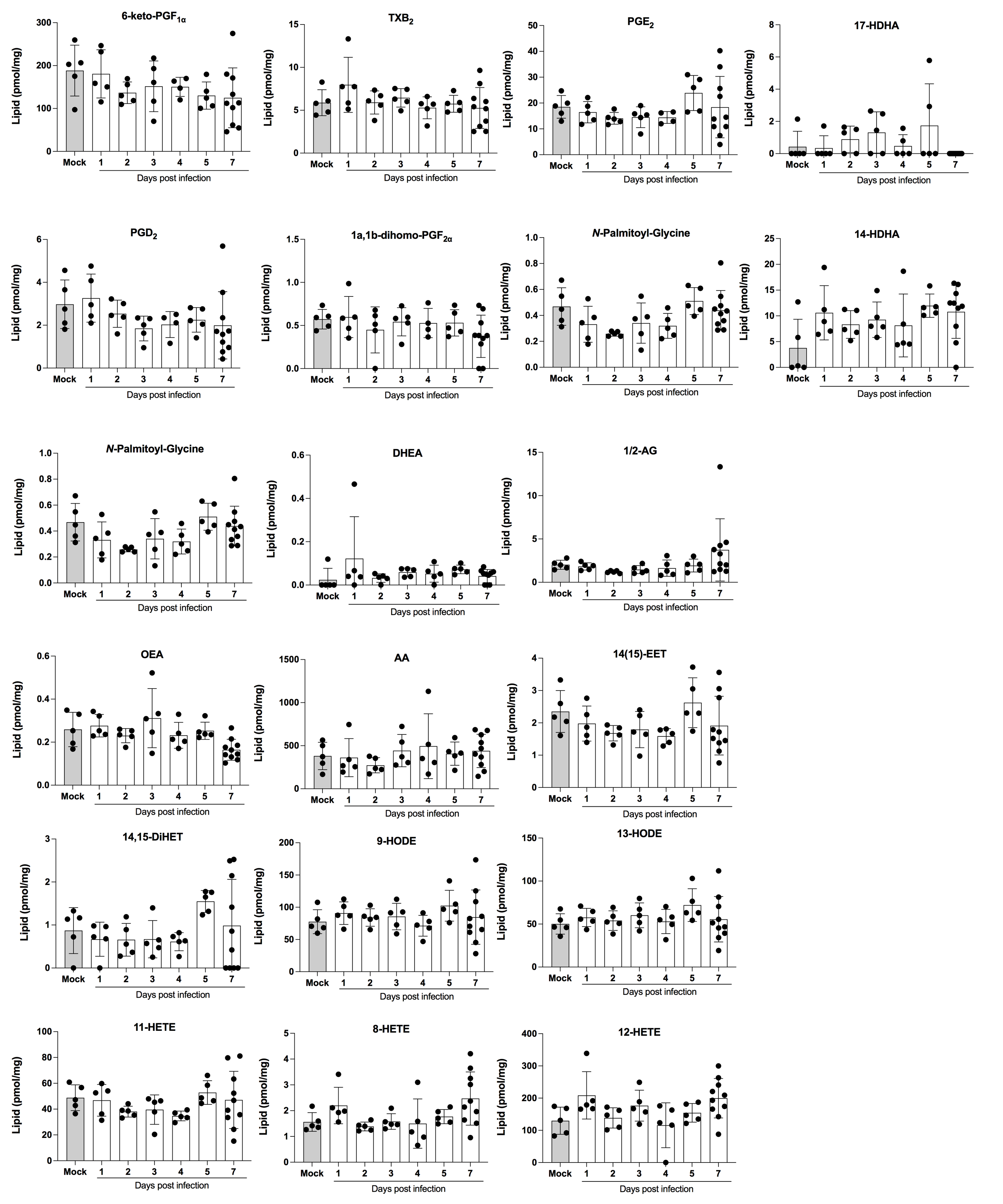

Supplement: Supplementary Figure 1 — Main lipid mediators that are unchanged in lungs of SARS-CoV-2 infected mice. Lung homogenates corresponding to 10 mg of tissues were heated at 60˚C for 30 minutes in presence of 1 volume of methanol containing deuterated internal standards. Samples then were processed as in Archambault et al (11) to extract the lipid mediators and were analyzed by LC-MS/MS using a previously described analytical method (15). P values were determined using the Kruskall-Wallis test, with p values <0.05 considered statistically significant. [file Image_1.tiff]
