# Supplementary material for: Cytokines and Lipid Mediators of Inflammation in Lungs of SARS-CoV-2 Infected Mice
Source: Front Immunol. 2022 Jun 24;13:893792. doi: 10.3389/fimmu.2022.893792 (PMC9264370; doi:10.3389/fimmu.2022.893792)
Supplement: Supplementary file 2 [file Table_1.pdf]

Supplementary Table 1: List of the top 20 upregulated genes during SARS-CoV-2 infection

Top 20 Upregulated genes								
Day 3			Day 5			Day 7		
symbol	log2FoldChange	padj	symbol	log2FoldChange	padj	symbol	log2FoldChange	padj
Gm11639	8,95	4,20E-14	Gm11639	8,67	5,92E-13	Kcnt1	8,52	2,66E-09
Ifnl2	8,72	8,51E-14	Efcab3	8,36	2,65E-11	Ankrd2	8,47	2,04E-06
Ifnb1	8,61	2,91E-13	4930438A08Rik	7,27	2,16E-08	Ngp	8,15	4,29E-10
Efcab3	8,38	1,32E-11	Cxcl10	6,93	1,08E-38	Cxcl9	7,62	2,35E-43
Cxcl10	8,34	1,78E-56	Gpr31b	6,85	1,75E-06	4930438A08Rik	7,55	2,85E-09
Gpr31b	7,56	5,56E-08	Cxcl9	6,52	6,44E-32	Agxt	6,57	2,37E-05
Cxcl11	7,52	3,21E-27	Ifnl2	6,41	2,38E-07	Cxcl2	6,45	2,25E-09
Acod1	7,52	4,39E-31	Ifnb1	6,41	3,04E-07	Gm8116	6,29	5,63E-03
Ccl7	7,39	4,98E-32	Cngb3	6,33	9,22E-14	Gpr31b	6,29	8,24E-06
Gm6445	7,37	3,48E-09	Ccl7	6,33	3,06E-23	Acod1	6,27	2,17E-21
Ifnl3	7,13	1,17E-08	Kcnt1	6,25	4,36E-05	Saa2	6,14	6,78E-04
Lhx2	7,06	2,03E-08	Dmbt1	6,24	2,96E-06	Gm4948	6,12	3,22E-04
Gm5960	7,02	6,96E-11	Cxcl11	6,23	2,16E-18	1700086D15Rik	6,12	5,85E-04
4930438A08Rik	6,99	5,45E-08	Acod1	6,17	1,13E-20	Cd300ld4	6,08	1,27E-05
Cxcl9	6,99	4,15E-37	Saa3	6,06	7,31E-15	Cxcl10	5,86	1,56E-27
Dmbt1	6,98	6,80E-08	Gm28437	5,96	1,67E-04	Drd4	5,82	2,19E-07
Gcg	6,96	1,92E-08	Oas1e	5,79	3,80E-06	Oas1e	5,80	1,89E-06
Rsad2	6,81	#######	Ifng	5,56	7,37E-09	Ubd	5,72	9,48E-13
Mx1	6,43	4,35E-93	Krt16	5,37	5,69E-05	Gzmc	5,70	6,25E-06
Trim30c	6,42	7,87E-22	Fgf23	5,31	1,04E-03	Ifng	5,62	2,73E-09

IFN-related genes

Cytokine/chemokine-related genes
