# Supplementary material for: Cytokines and Lipid Mediators of Inflammation in Lungs of SARS-CoV-2 Infected Mice
Source: Front Immunol. 2022 Jun 24;13:893792. doi: 10.3389/fimmu.2022.893792 (PMC9264370; doi:10.3389/fimmu.2022.893792)
Supplement: Supplementary file 3 [file Table_2.pdf]

Supplementary Table 2: List of the top 20 downregulated genes during SARS-CoV-2 infection

Top 20 Downregulated genes								
Day 3			Day 5			Day 7		
symbol	log2FoldChange	padj	symbol	log2FoldChange	padj	symbol	log2FoldChange	padj
Myl2	-17,32	1,07E-08	Cyp1a1	-5,67	8,09E-15	Cyp1a1	-7,90	3,25E-27
Cyp1a1	-5,21	7,06E-13	Prtg	-4,30	4,90E-03	S100a7a	-7,30	4,34E-07
Igkj3	-3,91	5,89E-03	Isl1	-3,97	5,24E-03	Gm10401	-6,82	2,48E-07
Gm15429	-3,69	1,91E-03	Cd209d	-3,80	2,57E-04	Rtl3	-6,32	1,89E-04
1700030C10Rik	-3,67	4,59E-03	Snx31	-3,73	8,71E-03	Slc5a9	-6,27	1,50E-04
1700016D06Rik	-3,34	7,47E-03	Abcc6	-3,35	1,35E-03	Cd207	-5,94	5,08E-07
Cyp26b1	-3,25	4,18E-08	Igkv17-121	-3,26	2,90E-03	Zfp385c	-5,37	1,44E-05
Ccl21d	-3,13	7,93E-03	Igkv1-135	-3,16	6,64E-04	Fabp1	-5,33	1,37E-16
Fabp1	-3,08	3,03E-11	Ces1g	-3,11	1,12E-05	Pcdhac1	-5,18	9,37E-04
Tepp	-2,95	1,39E-04	Hepacam2	-3,10	3,32E-13	Gkn3	-5,18	3,34E-09
Vstm2b	-2,64	7,82E-05	Fbn2	-3,07	1,46E-03	Bpifa2	-5,02	1,96E-05
Septin3	-2,59	3,95E-14	Cyp26b1	-3,05	4,84E-07	Sost	-4,89	9,56E-04
Vstm2a	-2,55	3,74E-05	Asgr2	-2,93	3,07E-03	Aplnr	-4,74	3,65E-47
Zim1	-2,54	8,71E-03	Colq	-2,92	2,73E-09	Gm7791	-4,54	1,11E-03
Abcc6	-2,54	8,94E-03	Ighj4	-2,87	1,01E-02	Ces2b	-4,54	8,93E-16
Colq	-2,45	5,43E-07	Tmprss5	-2,87	1,14E-03	Hes2	-4,48	1,19E-15
Cngb1	-2,36	6,26E-03	Fabp1	-2,83	9,99E-10	Gstm3	-4,31	1,34E-03
Skida1	-2,34	1,85E-19	Nmrk2	-2,77	8,14E-03	Gpr37l1	-4,30	1,13E-03
Fxyd2	-2,33	5,88E-05	Gpr141b	-2,73	4,77E-06	Cd209a	-4,20	5,19E-13
Ces1g	-2,31	1,11E-03	Lrat	-2,73	1,17E-10	Padi1	-3,98	2,87E-03
